# AMICAI: A Method Based on Risk Analysis to Integrate Responsible Research and Innovation into the Work of Research and Innovation Practitioners

**DOI:** 10.1007/s11948-019-00114-2

**Published:** 2019-06-13

**Authors:** Christopher Brandl, Matthias Wille, Jochen Nelles, Peter Rasche, Katharina Schäfer, Frank O. Flemisch, Martin Frenz, Verena Nitsch, Alexander Mertens

**Affiliations:** Institute of Industrial Engineering and Ergonomics, RWTH Aachen University, Bergdriesch 27, 52062 Aachen, Germany

**Keywords:** ELSA, ELSI, RRI, Technology assessment, Risk analysis

## Abstract

The integration of ethics into the day-to-day work of research and innovation (R&I) is an important but difficult challenge. However, with the Aachen method for identification, classification and risk analysis of innovation-based problems (AMICAI) an approach from an engineering perspective is presented that enables the integration of ethical, legal and social implications into the day-to-day work of R&I practitioners. AMICAI appears in particular capable of providing a procedural guidance for R&I practitioners based on a method established in engineering science, breaking down the object of consideration into partial aspects and prioritizing the innovation-based problems in dependence of potential risk. This enables the user to apply AMICAI continuously during all stages of the research and development (R&D) process and to analyze and choose between certain sociotechnical alternatives. In this way, problems that affect ethical, legal, and social aspects can be understood, reflected and considered in the mostly technically focused R&D process. The paper gives a general guidance about AMICAI by describing principles and assumptions, providing the steps of analysis and application aids, giving an example application, explaining the necessary adjustments of AMICAI compared to the methodical basis of failure mode, effects, and criticality analysis and discussing the advantages and limits. AMICAI’s simple applications can stimulate interdisciplinary cooperation in the R&D process and be a starting point for the development of an “open RRI risk analysis platform” allowing society to evaluate innovation-based problems.

## Introduction

Innovations are often the result of research and development (R&D) processes, which are typically carried out in small to medium project teams. The given objective is usually to solve a (technical) problem by applying or developing technology. The introduction of innovations is therefore often driven by technology rather than societal need, which has often been described as performing experiments on society for the main reason that effects of innovations are usually hard to predict (Kroes 2016). However, this approach leads to the challenge that undesirable effects are just detected towards or at the end of an innovation’s R&D process. From an engineering perspective, the analysis of undesirable effects in an R&D project of technical systems and products is usually limited to technical failures and their causes, for example due to the use of standard engineering methods such as failure mode and effects analysis according to IEC 60812 (2018). Aside from this focus on the innovation itself (e.g. functionality, reliability, and safety), the interaction between the innovation and the stakeholders (e.g. environmental protection, protection of personal data, and social implications) has to be considered during the R&D process as well, to avoid further undesirable effects (Greenbaum 2015). These further undesirable effects may derive from the fields of philosophy, bioethics and technology assessment as well as ethical, legal, and social implications/aspects (ELSI/ELSA) as well as responsible research and innovation (RRI). An overview of the origin of these concepts with focus on basic idea, objectives and ideals is given by Zwart et al. (2014), who describe the evolution from the processors philosophy, bioethics and technology assessment to ELSI, the shift to ELSA and finally the introduction of RRI. According to Armin Grunwald (2011), RRI combines applied ethics, technology assessment as well as science, technology and society studies (STS) research. At this stage RRI may be described as an evolving concept, with confusion as to motivation, theoretical conceptualization and translation into practice (Owen et al. 2012). René von Schomberg (2013, p 19) provides in a vision of RRI a proposal for a definition and describes “*a transparent, interactive process by which societal actors and innovators become mutually responsive to each other with a view to the (ethical) acceptability, sustainability and societal desirability of the innovation process and its marketable products (in order to allow a proper embedding of scientific and technological advances in our society)*”. Burget et al. (2017) pointed out that this definition has become the major point of reference in administrative definitions coming from science policy makers and various funding agencies, while academic definitions still have a lack of clarity concerning its definitions and dimensions. Consequently, the theory and practice of RRI tend to diverge (Macnaghten et al. 2014). Among others, Ribeiro et al. (2017) argue that further clarification on what RRI has to offer in practice is still needed. From an engineering perspective, this must include the procedural guidance for R&I practitioners throughout the R&D process to enable reflection on their own—mostly technical or engineering—work. R&I practitioners are usually given their targets, whether in the scientific environment through e.g. politics, funding agencies and the hierarchy of research institutions, or in the industrial environment through management decisions. Furthermore, Forsberg et al. (2016) mention that conducting RRI assessment on a general level fails to address the main challenge to making the results applicable to daily practice. Thus, it is the aim of the present article to address this need for methodological RRI support for the day-to-day-work of R&I practitioners. In summary, a substantially narrower scope of ELSI/ELSA/RRI is covered by the paper, i.e. approaching the integration of RRI in the day-to-day work of R&I practitioners aimed at establishing analyses of undesirable effects of research and innovation as an integral part of the design process. A variety of methods are available for practicing ethics in R&I. The literature review by Reijers et al. (2018) provides an overview of 136 sources, 74 of which include the application or description of 35 currently available general methods. These general methods can be categorized into the following three main types according to the time of application during the R&D process (Reijers et al. 2018):

1. Ex-ante methods use foresight approaches or construct scenarios, so that at the beginning of R&D processes the ethical effects can be anticipated without specific design or application;
2. Intra methods integrate practical ethics during R&D processes so that ethical values can be translated into design requirements or specific design recommendations;
3. Ex-post methods use known ethical issues of known innovations to ethically reflect the innovation after the R&D process.

Although such a categorization is not necessarily entirely consistent, because individual methods cannot usually be assigned exclusively to one category (Reijers et al. 2018), a conclusion can still be drawn from it. Fundamentally, the currently available methods from the field of RRI are typically applied more or less exclusively in only one phase of the R&D process. Furthermore, to our knowledge, in the field there are no methods available with an opportunity to quantify and therefore to prioritize the undesirable effects of innovations. However, this is necessary to continuously consider undesirable effects during the day-to-day work alongside actual R&D work, because it enables to concentrate on those with the high risk. In this way the demand of Reijers et al. (2018) that approaches dealing with ethical technology design should focus more on the integration of ethics in the day-to-day work of R&I practitioners can be satisfied. The discussion of the individual approaches will be omitted at this point, as it does not represent the core of the present paper. Accordingly, from an engineering perspective, methods are required that (a) can identify and quantify undesirable effects of the innovation with its stakeholders and (b) can be continuously applied in the day-to-day work during all phases of the R&D process. For a persistent application, such methods have to be easy to use for the developers of the innovation, i.e. R&I practitioners, and should be able to serve RRI. A method as previously stipulated must break down the object of consideration into partial aspects so that changes can be incorporated and evaluated quickly. In addition, it must be possible to prioritize the investigated implications of an innovation so that limited resources of R&D projects can be effectively applied to the most important or most critical ones. The quantification of manageable partial aspects and the subsequent merging of the partial results is necessary for this purpose. In this way, the growing knowledge about these undesirable effects can be continuously explored step by step from the beginning of R&D projects. Thus, modification of the innovation or changes in ethical standards, legal requirements, or social values can also be taken into account on the fly as they occur. In order to address these requirements, the application of the methodology of risk analyses or assessments in the area of RRI may be effective, as risk analyses are already an integral part of R&D process. On the basis of the studies by Chatfield et al. (2017) on the high relevance of risk assessment, including the consideration of ethical and social issues, it is suggested that employing the theoretical lens of risk in a pragmatic “first step” approach can be a familiar language to companies and thus R&I practitioners. However, the area of risk analysis within RRI covers only the classic safety topics by avoiding (technical) product failures with a focus on potential hazards (Agapito-Tenfen et al. 2018; Beaudrie et al. 2014; Forsberg et al. 2016; Van Wezel et al. 2018). Accordingly, it is proposed to extend the methodology of traditional risk analysis to the field of RRI and in particular be able to take also ethical, legal and social effects into account in the analysis.

The failure mode and effects analysis (FMEA) according to the standard IEC 60812 (2018) is a systematic standard method in the field of engineering for evaluating an item or process to identify the ways in which it might potentially fail and the effects of the mode of failure upon the performance of the item or process and on the surrounding environment and personnel. The fundamental principles in engineering science indicate that the cost and effort of change—e.g. to eliminate a technical failure—increases exponentially with progression in development and design of technical systems and products (Chaffin 2005). FMEA was designed in such a way that it can already be used for failure detection in the early stages of the R&D process. For its purpose, FMEA is a standard method, frequently used during all stages of R&D processes as an analysis technique for determining and quantifying possible failure modes and their causes and effects on the behavior of the system (Doshi and Desai 2017). When failure modes are prioritized, the process is referred to as failure modes, effects, and criticality analysis (FMECA), taking into account the combination of a failure’s severity, probability of occurrence, and/or detectability (IEC 60812 2018). FMECA is a promising approach to transfer a method already established in engineering science into the RRI context. In this way, the analysis of criticality is included in the proposed method. This is supported by statements of the IEC 60812 (2018) itself, which explicitly states that FMECA can also be applied outside of an engineering R&D process. Accordingly, FMECA provides a general framework for the technical reliability of systems or processes by analyzing failures that basically meet the previously specified requirements for the field of RRI. Consequently, a method based on the FMECA was developed which is to be used by R&I practitioners in day-to-day work and thus provides the basis for continuous consideration of RRI aspects during technology development.

The proposed method was developed and piloted extensively at RWTH Aachen University and is known by the acronym AMICAI. The acronym stands for “Aachen method for identification, classification and risk analysis of innovation-based problems”, named after the city in which it was developed. The AMICAI approach tries to close the methodological gap shown by Reijers et al. (2018) with regard to the integration of ethics into the day-to-day work of R&I practitioners, which means that the access and feasibility of the method is largely simple for users who are not deeply involved in the RRI. AMICAI is chosen as the subject of the following descriptions, based on the requirements of such a method as described before. The paper gives a general guidance about AMICAI by:

- Describing principles and assumptions,
- Providing the steps of analysis and application aids,
- Giving an example application,
- Explaining the necessary adjustments of AMICAI compared to FMECA and
- Discussing the advantages and limits.

## Method of AMICAI

AMICAI offers a method for the continuous analysis of innovation-based problems during the R&D process from the very early stages. AMICAI is used to analyze problems of subsequent use or application of the innovation. This involves identifying and classifying problems and prioritizing them by means of a risk analysis. The following description refers to the implementation of AMICAI from scratch, usually at a very early stage of the R&D process of an innovation.

**Fig. 1:**
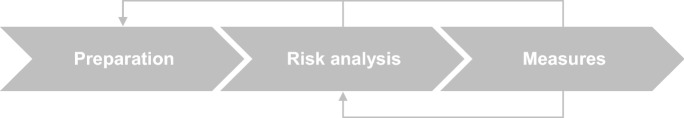
Phases of the application of AMICAI

The application of AMICAI is divided into the three phases of preparation, risk analysis, and measures (Fig. 1). However, the main methodological improvement with AMICAI comprises risk analysis of undesirable effects of the innovation with its stakeholders, which are operationalized in the application of AMICAI as problems, their causes, and their effects. In accordance with the FMECA methodological model, the severity, probability of occurrence, and detectability of the problem are determined. By mathematically combining these factors, a risk priority number can be calculated for each problem to create a ranking order. Achieving a same understanding of terms within the application of AMICAI, they are defined in “Appendix 1”. A process diagram of AMICAI including all relevant steps is given in “Appendix 2”.

### Preparation Phase

For the preparation phase, a processing of the following subsequent steps is proposed.

1. Define objectives and scope of analysis.
2. Define the viewing context and quantification scales.
3. Define classification.
4. Select analyst.

The application of AMICAI requires a clear objective and scope to ensure that the results are of sufficient extent and accuracy. In particular, the analysis effort has to be determined, e.g. the number of persons involved and the frequency of repetition of the application of AMICAI. The definition of the viewing context includes at least a short description of the envisaged innovation with functions and characteristics as well as the later application. Abusive applications or all possible applications of the innovation should not be included in the viewing context, because they can be identified as problems in subsequent steps of the application of AMICAI. The quantification scales should be defined in appropriate granularity to the viewing context. In Sect. 2.2, generally applicable scales are shown. Defining a classification as the next step includes the definition of stakeholders and viewing levels. The stakeholder is conceptualized as an individual or a group of people directly or indirectly affected by the envisaged innovation. A stakeholder can also not be a natural person (e.g. nature or law) represented by legal entities or interest groups. All stakeholders within the classification should be chosen for the purpose of integrating different perspectives. Stakeholders who are affected in a similar manner by the envisaged innovation can also be brought together. The viewing levels conceptualize areas in the fields of ELSI, ELSA, or RRI to which usually several problems can be assigned. The definition of viewing levels can be based on existing structure models of these areas or on the case-specific development of a structure model of the areas. The viewing levels must be formulated in a value-neutral way. Once the classification has been defined, the analysts of AMICAI must be selected. The analyst either represents a stakeholder or has expertise in one or more areas of the viewing levels. An analyst can also fulfil both of these characteristics. The number of analysts should be based on the objectives and scope of analysis. For example, when AMICAI is applied once at the beginning of the R&D process, it makes sense to focus on the most important stakeholders and viewing levels with a manageable number of analysts.

### Risk Analysis Phase

The risk analysis phase comprises the identification and quantification of problems. This will ensure an effective and efficient working method in the application of AMICAI, because the identified problems will be quantified immediately. Application aids are available for the risk analysis phase (“Appendix 3”). For the second phase, the processing of the subsequent steps is proposed.

5. Identify problems and assign to classification.
6. Identify problem effects.
7. Quantify severity (S) of problem effect.
8. Identify problem causes.
9. Quantify probability of occurrence (O) of causal chain.
10. Identify detection methods.
11. Quantify detectability (D) of problem cause.
12. Calculate the risk priority number (RPN).
13. Prioritize causal chains.
14. Determine critical causal chains.

**Fig. 2:**
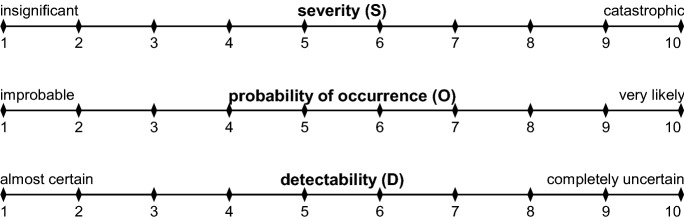
Quantification scales of severity, probability of occurrence, and detectability

A problem is defined as a possible undesirable effect or condition in the viewing level of a stakeholder due to the envisaged innovation. The problems can be identified either systematically by given classification or unsystematically without a given classification. In the case of unsystematic identifying of problems, the classification is gradually evolving. Of course, existing classifications can also be extended or adapted when identifying new problems. The problem effects are then identified and quantified immediately in terms of their severity (S). In the next step, the problem causes are identified. This completes the causal chain as a relationship between the problem cause, the problem, and the problem effect, which can now be quantified according to the probability of occurrence (O). The intended detection method is the method by which a problem of an innovation becomes evident by detecting the problem cause. The detectability (D) of the problem cause by means of the intended detection method shall be quantified. The scales shown in Fig. 2 have proven to be practical for quantifying the severity (S), the probability of occurrence (O), and the detectability (D). If inappropriate for quantification, the scales may also be changed. However, it should be noted that different scale sizes lead to a previously unintentional weighting of the three factors to be quantified. To this end, for each problem the three quantified factors are multiplied to calculate the risk priority number (RPN). Therefore, the RPN represents a combination of severity, probability of occurrence, and detectability of a single problem.

Once the complete causal chains have been identified and quantified, they must be prioritized. For prioritization, the RPNs are sorted in descending order, based on the understanding that higher RPNs are more critical than lower RPNs. This helps in determining which causal chains are critical, so that they are focused on in the following measures phase. Causal chains that have not been classified as critical are set “on hold”. Depending on changes in innovation or the framework conditions, these can become objects of observation again later on. It is also conceivable that causal chains with high RPNs are set “on hold”, because it is assumed that the problem is volatilized by the R&D process.

### Measures Phase

The measures phase is carried out on the basis of the identified problems, quantification of severity, probability of occurrence, and detectability as well as the prioritization by RPN. The measures phase starts with thoughts about possible problem compensation measures conducted with the aim of identifying first approaches to eliminating or reducing the impact of the problem cause and the expertise needed to be involved. AMICAI usually only prepares the measures phase because the measures for adapting the innovation have to be developed and implemented by specialists and afterwards reflected back for reanalysis. For the measures phase, no generally applicable procedure can be proposed because the process needs to be tailored to specific needs and depends on the required expertise. However, below are listed some methods that are applicable in the measures phase. Once a suitable solution has been found for a problem compensation measure, the risk analysis phase of the problem has to be repeated as there may have been changes in the problem or its quantification.

## Example Application of Risk Prioritization

Digitization is leading to a major upheaval in working environments and its effects have not yet been fully investigated. Accordingly, the following innovation from the field of future production work is an excellent example application for AMICAI.

**Fig. 3:**
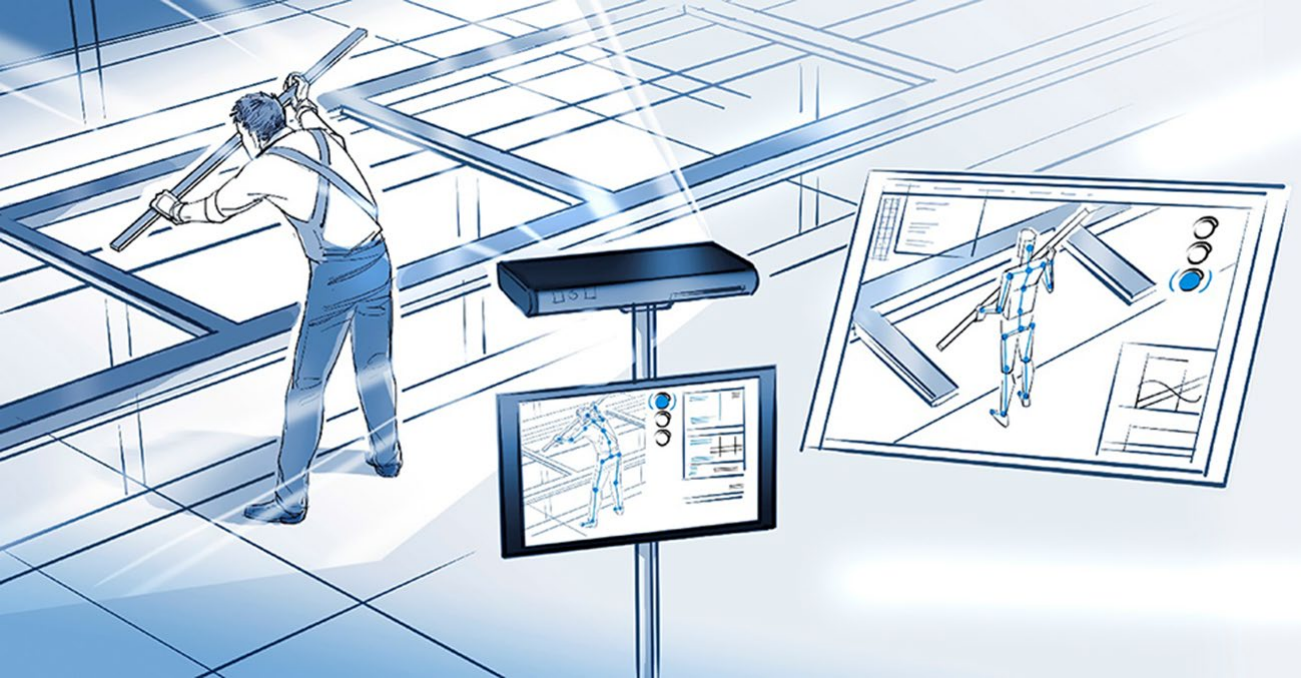
Innovation sketch for example application of AMICAI

Despite increasing automation, people will continue to form an inherent part of the socio-technical production system, which is why the ergonomic design of workplaces, processes, and resources will remain of great importance. Digitization opens up completely new potential, e.g. for an automated ergonomic risk assessment of work processes in real time and associated short-cycle feedback for the employee. Such a system would integrate time-of-flight sensors for motion capture and appropriate data processing for automated execution of ergonomic risk assessment methods and thus allow a direct derivation of corrective measures (Brandl et al. 2016). To illustrate the idea, Fig. 3 shows a sketch of the innovation. Such a system could be capable of solving current fundamental problems of ergonomic risk assessment, such as the low reliability of evaluations because of subjective influences and the high effort of manual data acquisition and evaluation (Brandl et al. 2017a). Through the reduced effort, employees can be given individual feedback on their patterns of movement and behavior, which is advantageous over the current collective strategy (Brandl et al. 2017b).

**Fig. 4:**
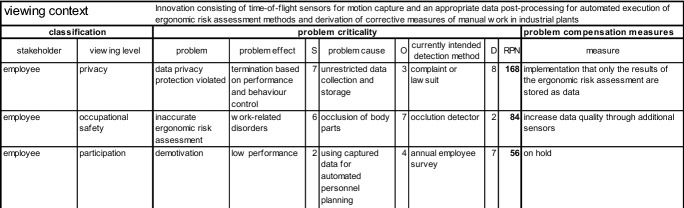
Excerpt of three problems analyzed within the example application of AMICAI

AMICAI was conducted by a group of different analysts consisting of an employee, a company physician, an executive manager, an ergonomist, an occupational health and safety specialist, an ethicist, a sociologist, and a counsellor. The example application of AMICAI shows an excerpt of the three problems “inaccurate ergonomic risk assessment”, “data privacy protection violated”, and “demotivation” to especially demonstrate the procedure for risk prioritization. The classification shows that employees are affected as stakeholders in the three viewing levels “occupational safety”, “privacy”, and “participation”. Figure 4 shows the documentation of AMICAI results of the three problems considered using the form sheet.

The problem of “inaccurate ergonomic risk assessment” can cause “work-related disorders” for an employee. The severity of the problem effect “work-related disorders” was rated by the analysts with a medium severity of S = 6 on the defined ten-point scale. The most likely problem cause is assumed the occlusion of body parts during measurement. The causal chain is quantified with a probability of occurrence of O = 7 on the defined ten-point scale, as such occlusions are quite frequent in crowded industrial plants. At this stage it is unclear whether the problems caused by occlusion can be solved technically. Accordingly, it is to be assumed that occlusion will occur. This makes it necessary to recognize occlusion when it occurs. A detection method is to be implemented that shows the later user which parts of the body were occluded during the measurement. A video image is stored for a short time before and after the occurred occultation to check the data quality and for approval by the user. With this detection method, it is to be assumed that the problem cause can be reasonably well detected and the problem effect can be avoided. The detectability was correspondingly rated by the analysts as certain with D = 2 on the defined ten-point scale. The risk priority number is the product of the three quantified aspects and results in S · O · D = RPN = 84. The described procedure is repeated accordingly for the other three problems and produces the results shown in Fig. 4. As a second problem, “data privacy protection violated” has been identified, which can result in termination based on performance and behavior control. The severity of this problem effect was rated by the analysts with a medium to high severity of S = 7, because the termination of the work contract is a negative effect for the employee. The most likely problem cause is assumed to be the unrestricted collection and storage of data. The causal chain is quantified with a probability of occurrence of O = 3, because it is possible to extract data for performance and behavior control at the current R&D stage of innovation. The problem will probably only be detected during later use when a complaint or lawsuit is filed. Accordingly, the problem or its effect is only detected once it has occurred. The detectability is therefore not preventive and was therefore assessed as rather uncertain and rated D = 8. The risk priority number for the problem “data privacy protection violated” is RPN = 168. With “demotivation” and the possible effect of “low performance” a third problem was identified, which could most likely be caused by using the captured data for automated personnel planning. This is because employees are not able to participate in decisions on personnel planning, as is usually the case, but are assigned using the data-based algorithm. The severity of the problem effect was rated with S = 2 as nearly insignificant. The analysts found the probability of occurrence to be possible with O = 4. The current intended detection method is an annual employee survey, which is expected to detect the problem after it has already occurred. The detectability was rated with D = 7. The risk priority number was calculated with RPN = 56. The three causal chains are sorted in descending order according their RPN, as recommended by AMICAI and shown in Fig. 4.

In this example application of AMICAI, the problem “data privacy protection violated” was identified as the highest risk for an innovation-based problem. As a measure, it was determined that only the results of the ergonomic risk assessment can be stored as data and unrestricted data storage is avoided. This data alone would make it virtually impossible to monitor performance and behavior. For the problem “inaccurate ergonomic risk assessment”, it was determined as a measure that the data quality should be improved by using additional sensors in order to eliminate the problem cause. The problem “demotivation” was set “on hold” for later reanalysis. The two problems of interest have to be passed on to the R&D process for consideration of the problems and to implement the problem compensation measures. After implementing the measures as innovation changes, the problems are reanalyzed. This procedure allows innovation-based problems to be analyzed continuously during the R&D process, changes in innovation or framework conditions to be taken into account and the currently critical problems to always be indicated.

## Notes for the Successful Application and Adaption of AMICAI

AMICAI offers a general method for the analysis of innovation-based problems. A necessary criterion for the application of AMICAI—independently of any modifications made—is the moderation by an experienced moderator trained in AMICAI. The moderator’s task includes in particular the proper use of the defined terms, thus ensuring consistency in the use of the terms. For the application of AMICAI to be successful, appropriate resources for teamwork must be allocated. A precise understanding of the (technical) functioning of the innovation to be analyzed is not necessary for the application of AMICAI, but a clear understanding of the viewing context is. However, as the R&D of an innovation progresses, detailed analysis requires in-depth knowledge of the innovation features and their specifications. Manageable R&D work usually requires the integration of expertise from several areas, which is why sufficient resources are required. As AMICAI also includes the methodology of detection of potential problems, it is furthermore important to have at least some competence in judging appropriate methods within the team. Furthermore, it should also be pointed out that teamwork benefits fundamentally from diversity.

The method of AMICAI can be adapted to the most varied conditions of application, whereby in most cases a change in the sequence of the steps is sufficient. If an innovation is to be analyzed from scratch with a trained moderator, the method in the intended order described above is well suited. However, if there is little experience in using AMICAI, it is advisable to carry out the intermediate step of exploring problems before developing the classification.

In general, a classification can exist before problems are identified or a classification is developed based on identified problems. An existing classification can always be extended or modified based on the results of following steps. However, the granularity of the classification should always be adapted to the status of the R&D process of the innovation, so that AMICAI remains manageable for the analysts. For the analysis of innovations in early R&D stages, therefore, a classification of low granularity is usually recommended, initially limited to the directly affected stakeholders and generally formulated viewing levels. Depending on the expected extent of the application of AMICAI (e.g. a very radical innovation), it may make sense to divide the classification into independent areas and to carry out AMICAI several times. This reduces the complexity of implementation for the analysts, which usually results in a better quality of results.

For the risk analysis phase, it is also possible to deviate from the sequence of steps shown. In general, it is advisable to carry out a risk analysis one after the other for each problem. However, it is also possible to perform each step completely for all identified problems and then proceed to the next step for all identified problems. Furthermore, it can sometimes make sense to identify and analyze the problems of one viewing level and then proceed with the next viewing level. With an increasing number of problems, risk analysis activities should be focused on areas of classification where few or no problems have been identified so far. In general, it does not matter whether the problem, the problem cause, or the problem effect is identified first, as long as these are correctly classified and incorporated into the causal logic of AMICAI. Specifically, this means that it is only important to differentiate consistently between problems and their causes and effects, while always starting the analysis with the problem. For this purpose, a chronological-causal connection, the so-called causal chain, was introduced to AMICAI. It can also occur that causal chains of problem causes, problems, and problem effects continue or overlap due to different stakeholders or classifications.

## Discussion

Zwart et al. (2014) point out that RRI no longer sees the ethical aspects of new technologies as constraints or restrictions. Instead, the aims of technology development and the positive contributions from R&I should be considered (Zwart et al. 2014). A rubric for assessing RRI proposed by Wickson and Carew (2014) can be used as an example of how RRI can be used to transform to a positive analysis. It is understandable that this positive presentation promotes the general acceptance and thus the consideration of RRI when defining R&I targets. However, the undesirable effects of research and innovation cannot be ignored and must therefore be part of RRI. The importance of problem-orientation in emerging science and technologies assessment is, for example, discussed by Forsberg et al. (2016). Chatfield et al. (2017) concluded that employing the theoretical lens of risk in a pragmatic “first step” approach can be a familiar language to companies and thus R&I practitioners. Now it may be a good compromise that RRI focus more on positive contributions where risks and concerns can quickly become an obstacle to research and innovation on the one hand. On the other hand, focusing on the analysis of undesirable effects in day-to-day work of R&I practitioners may lead to RRI becoming an integral part of the design process. This can be done following the example of risk analysis for product safety, which is an integral part of R&D. This compromise, however, is in line to the statements made by Forsberg et al. (2016), which stated that there is a difference between the bird’s eye view and to really zoom in on the details to make the results applicable to daily practice. This difference must also be reflected in the methodological support and somehow be brought together. It is therefore also a question of weighing the value and risk of an innovation, which, as Stahl et al. (2014) describe it, can initially end in an ethical dilemma that needs to be resolved. However, the AMICAI approach appears to be a promising way to providing procedural guidance for R&I practitioners on how to analyses undesirable effects of research and innovation as an integral part of the design process.

In accordance with the literature review by Reijers et al. (2018) on methods for practicing ethics, from an engineering perspective it can be argued that a particular focus should be placed on the integration into day-to-day work of R&I practitioners. AMICAI appears capable of improving this issue by (1) providing a procedural guidance based on a method established in engineering science, (2) breaking down the object of consideration into partial aspects, and (3) prioritizing the innovation-based problems in dependence of potential risk. This enables the user to apply AMICAI continuously during all stages of the R&D process and to analyze and choose between certain sociotechnical alternatives. In particular by the possibility to divide the problems into partial aspects, AMICAI offers an easy way to extend or repeat the analysis, e.g. in the event of changing framework conditions or innovation characteristics. In this way, problems that affect ethical, legal, and social aspects can be understood and considered in the mostly technically focused R&D process. The early application of AMICAI will ensure a process-oriented and relatively inexpensive mitigation of innovation-based problems. AMICAI stimulates the interdisciplinary cooperation between different disciplines in R&D. In addition, the approach provides an overarching and fundamental understanding of innovation-based problems in the sense of risk analysis. This means that for the identified problems the risk is always determined for the aim of minimizing it and with the understanding that there will always be a residual risk.

The proposed method has its methodological origin in FMECA, a method established in engineering science for the analysis of usually technical-constructive problems. Based on FMECA, AMICAI was adapted accordingly to the objectives and scope of an analysis of innovation-based “non-technical” problems. AMICAI provides a procedure for identifying, classifying, and prioritizing innovation-based problems during all phases of the R&D process. However, AMICAI offers connection points for the integration of existing methodological approaches and tools, which are described below. Limitations or shortcomings of FMECA described extensively in ICE 60812 (2003) apply to AMICAI in a similar manner. However, due to the adjustments made, there are differences in the limitations which will be discussed below, and from which a demand for future work can be derived.

### Integration of Existing Methods and Tools

This section shows examples of where other concepts and methods can be integrated into the application of AMICAI. AMICAI offers a generally applicable procedure in the proposed form, in which various approaches can be integrated or exchanged for partial aspects. The overview is not intended to be exhaustive, but merely to demonstrate that other methods can be easily integrated into the application of AMICAI. Depending on the objective and scope of analysis, methods which are not shown here in combination with AMICAI may lead to even better results.

The preparatory phase can be supported by other methods, in particular with the definition of the viewing context and the classification as well as the selection of stakeholders. The scenario planning (Boenink et al. 2010) offers an excellent opportunity to develop methodically the later application of the innovation for the viewing context. The research about MEESTAR of Arne Manzeschke (2015) or the standard VDI 3780 (2000) of the association of German engineers can be used as a basis for classification. Furthermore, ethical checklists as proposed by Brey (2012), Stahl (2011), and Palm and Hansson (2006) or frameworks as proposed by Heintz et al. (2015) and Wright (2011) can be used to find the areas of interest and thus define the classification. For the selection of stakeholders Yves Fassin (2009) and Achterkamp and Vos (2008) provide methodical support. However, for methods that deal with stakeholder identification, Reijers et al. (2018) demand a justified stakeholder selection.

The risk analysis phase—especially the identification of the problem—can be easily supported by well-known creativity techniques, such as brainwriting, mind mapping, or thinking outside the box. Furthermore, it is possible to support the quantification of the three factors by detailing the scales shown in Fig. 2, as provided for example in IEC 60812 (2003). However, while doing so, every scale should be treated the same way to avoid unintended overemphasis of one scale. The calculation of the common form of the RPN—multiplication of severity, probability of occurrence, and detectability—is probably the procedure most often used to analyze the criticality. However, ICE 60812 (2003) gives further opportunities for criticality analysis methods, such as criticality plots (providing simple plots of probability of occurrence against severity with criticality ranks being assigned according to bands within the plot), criticality matrix (providing a matrix of probability of occurrence and severity with criticality ranks being allocated to each of the cells within the matrix) as well as the alternative RPN (providing a more consistent analysis of criticality when parameters can be quantified on a logarithmic scale).

The measures phase can usually be supported by well-known creativity techniques, as pointed out for the identification phase and problem-solving methods such as Theory of Inventive Problem Solving (TRIZ) (VDI 4521-1: 2016). Souder and Ziegler (1977) provide a still valid overview that can be used for a selection of suitable methods. However, as pointed out in the previous section, the procedure in this phase depends on the required expertise. It is evident that for problem compensation measures which require the development of a technical-constructive solution, different methods and procedures are necessary than for the development of software solutions or the adaptation of business models.

The main improvement by AMICAI in the analysis of innovation-based problems is the implementation of a risk analysis for quantification and prioritization. Thus approaches and methods of technology assessment (TA), ethical technology assessment (eTA), constructive technology assessment (CTA), ethical-constructive technology assessment (eCTA), and science and technology studies (STS) as reviewed for example by Kiran et al. (2015) and Palm and Hansson (2006) can be extended by AMICAI or used as an extension of it.

### Limitations and Further Work of AMICAI

With regard to RRI, the present description of AMICAI initially provides a procedural guidance for the day-to-day work of R&I practitioners at process level. Precise explanations and empirical evidence at product level must be the subject of further research. The limitations of FMECA also apply to AMICAI. AMICAI can be a work-intensive and inefficient process if it is not used wisely. Therefore, in particular, the objectives and scope of analysis should be determined and AMICAI should not be included indiscriminately in requirement specifications.

From a methodological point of view, however, quantifying and prioritizing the innovation-based problems is not an easy task, otherwise numerous methods would have been established in the day-to-day work of practitioners. With the analytic hierarchy process, another quantifying and prioritizing methodology for prescriptive decision-making has already been proposed, which might be an easy and useful tool for application in the field of RRI (Monsonís-Payá et al. 2017). Quantification in general and as defined by AMICAI, for example, entails, in particular, uncertainties resulting from over- and underestimation of the assigned numerical values. This issue is methodically relativized by splitting the quantification into partial aspects, which leads to a reduction in complexity. However, it is important to investigate the reliability of quantification, for example depending on different group compositions, and to deduce possible indications of the “ideal” composition of analysts. Another important point is that risks can be objectified and thus appear objective, despite the largely subjective nature of the topic. Using AMICAI as an easy-to-use method in combination with a web-based platform may enable society to evaluate innovation-based problems. If then a multitude of data from a wide range of stakeholders can be collected in quantifying these problems, the field of objectivity, validity and reliability of quantifying risk analysis may also be better investigated and understood based on empirical data.

Shortcomings of the risk priority number are widely known and were clearly summarized in a review of 75 studies by Liu et al. (2013). The calculation of RPN by multiplying the three factors severity, probability of occurrence, and detectability has been criticized often. For the quantification of the factors, ordinal scales are usually used. This makes it difficult to interpret the concrete value of an RPN, because for example an RPN twice as high does not therefore represent twice as high a risk, but a substantially higher risk. Furthermore, it is not guaranteed that similar risks are assigned the same RPN and it is possible that there are risks with the same RPN that are not equally acceptable. This effect is amplified when the interpreters of the results apply different weightings to the three factors severity, probability of occurrence, and detectability. In order to avoid misleading conclusions from RPN comparisons, which result from the fundamental fact that the scales are ordinal scales and not ratio scales, the RPNs are sorted in descending order when using AMICAI.

Another limitation of AMICAI is that the problems analyzed are not always independent of each other. FMECA is not suitable for considering dependent failures or failures resulting from a sequence of events (IEC 60812 2003). Since a failure mode can have more than one cause, it is therefore necessary to identify and describe the most likely possible, independent causes when using FMECA. This procedure seems impossible for AMICAI and has been adapted accordingly, because the causal relationships between the problem causes, problems, and problem effects cannot always be determined as clearly and directly as they are in technical systems (Karwowski 2012). For this reason, the application of AMICAI creates causal chains consisting of problem cause, problem, and problem effect. This partially resolves the methodological inadequacy of FMECA with regard to the analysis of dependent causes of failure. This not only quantifies the probability of the problem occurring, but also that of the entire causal chain, which means that possible dependencies can be taken into account. For example, the probability of the causal chain occurring with one problem cause is less quantified if it is known that a second problem cause must necessarily occur for the problem to occur. However, other methodological approaches, e.g. according to the standard IEC 60300-3-1 (2003), Xiao et al. (2011), or Zammori and Gabbrielli (2012), can be used to model the dependencies of problem causes for a more precise understanding and consideration. In addition, multi-criteria decisions can be supported, e.g. with approaches of Haimes et al. (2002) and Linkov et al. (2006). For early-stage assessment challenges of high uncertainty technologies, Linkov et al. (2018) propose a risk governance approach that integrates quantitative experimental information alongside qualitative expert insight to characterize and balance the risks, benefits, costs, and societal implications of emerging technologies, for example. But also interdisciplinary approach to the management of resilience seem to be useful for RRI (Naderpajouh et al. 2018) and further work of AMICAI. For the analysis of innovation-based problems the described procedure is sufficient to deliver good results. In contrast to purely technical failure analysis, the analysis of innovation-based problems according to concepts such as ELSI, ELSA, and RRI will always be somewhat blurred.

AMICAI extends the scope of existing RRI methods to support the integration of ethics into the day-to-day work of R&I practitioners, especially at the process level, and thus makes a contribution towards RRI. Accordingly, AMICAI can substantially support the risk analysis of innovations within concrete application scenarios. However, an overall assessment of global technological development trends and phenomena such as artificial intelligence, big data, block chain, brain-computer interface, digital twins, exoskeleton, and human–robot collaboration is not intended. One of the main tasks for the future will then be to reliably combine AMICAI with RRI methods. This combination can begin at the RRI process level and eventually extend to the product level. Furthermore, the ease of use of AMICAI makes it more accessible to the public in the sense of open innovation and to carry out a collaborative analysis of innovation-based problems of existing or planned R&D activities. For this, AMICAI would have to be implemented as an “open RRI platform”. This might also help to solve the fundamental problem of appropriate stakeholder participation and selection.

## Conclusion

In the present form, AMICAI provides a continuously applicable method in an innovation’s R&D process for the identification, classification, and risk analysis of innovation-based problems, which offers the opportunity of integrating several existing approaches and methods. AMICAI thus offers an approach to solving the demands of Reijers et al. (2018) for the integration of ethics into the day-to-day work of practitioners who are developing especially engineering innovations. Even if the method has been designed for application with engineers, it seems reasonable to assume that it will also apply to non-engineers developing innovations, such as business models or basic scientific research results in natural science. For a sustainable RRI, AMICAI can be used as a starting point for the development of an “open RRI risk analysis platform” in the sense of open innovation.

The development of AMICAI was carried out on the basis of FMECA. At least in Europe, directive 2001/95/EC of the European parliament and of the council requires the application of methods such as FMECA for general product safety. It therefore seems justified to call for this in the field of ELSI, ELSA, or RRI on the basis of the existing analogy. Such an analogous commitment to evaluate and demonstrate acceptable innovations from the perspective of ELSI, ELSA, and RRI allows the methods to be anchored in the R&D process in a sustainable manner. Against the backdrop of ever more complex innovations and ever shorter innovation cycles, this seems to be a necessary approach, because otherwise developers will have to consider these aspects less or not at all when developing innovations, primarily because of the decreasing time available.

## Figures and Tables

**Fig. 5:**
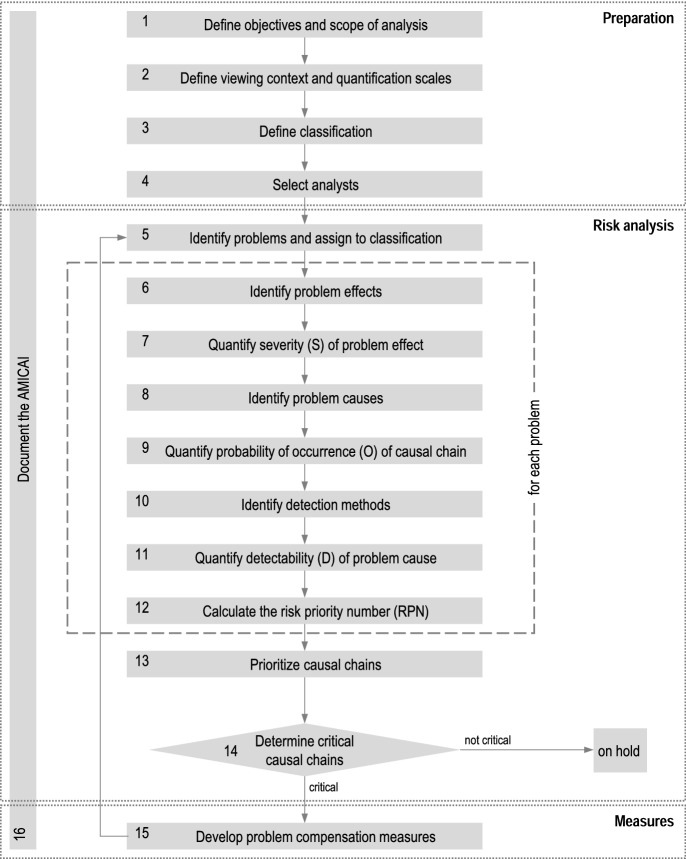
Process diagram for one cycle of application of AMICAI

**Fig. 6:**
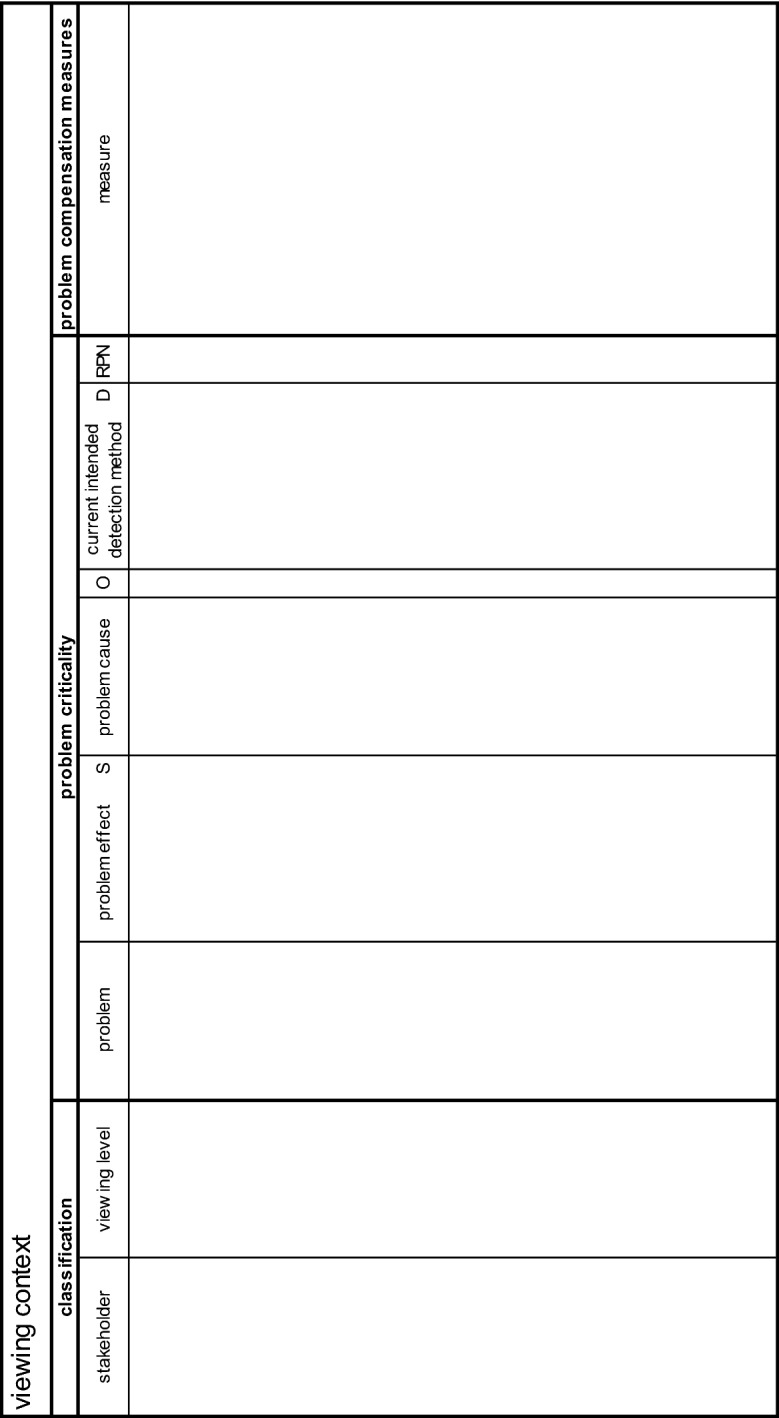
Form sheet for documentation of the results of AMICAI

## Appendix 1: Definition of Terms in Alphabetic Order

AMICAI is an acronym and stands for the “Aachen method for the identification, classification, and risk analysis of innovation-based problems”.

The analyst either represents a *stakeholder* or has expertise in one or more areas of the *viewing levels*. An analyst can also fulfil both of these characteristics.

The causal chain describes the temporal-causal cause-and-effect relationships between the *problem cause*, the *problem*, and the *problem effect*. The *problem cause* leads to the *problem*, which in turn leads to the *problem effect*. The same *problem* can have different *problem causes* or *problem effects*, which will lead to different causal chains. The three elements in the causal chain can be distinguished from each other by assigning the *problem cause* to the innovation, the *problem effect* to the *stakeholders*, and the *problem* to the direct interaction of innovation and *stakeholders*.

The classification distinguishes between *stakeholders* and *viewing levels*.

The detectability (D) is a relative ranking for the *problem cause* by the intended *detection method*. Detectability can be quantified on a scale ranging from almost certain (1) to completely uncertain (10). If the *problem cause* cannot be detected, a suitable detection method should be found for the *problem* or the *problem effect*. Accordingly, the detectability is to be considered more uncertain because the *problem* or the *problem effect* is not detected before it occurs.

The detection method is the method by which a *problem* of an innovation becomes evident by detecting the *problem cause*. It makes sense to consider only *detection methods* that will be applied in the later use of the innovation. A basic distinction can be made between detection methods which will detect the *problem cause*, the *problem*, or the *problem effect*. The focus should be on detection before the *problem* or the *problem effect* occurs.

The probability of occurrence (O) is a relative ranking for the *causal chain*. The probability of occurrence can be quantified on a scale ranging from improbable (1) to very likely (10). However, other quantification scales are possible.

A problem is defined as a possible undesirable effect or condition in the *viewing level* of a *stakeholder* due to the envisaged innovation, but also as concerns or reservations. A problem can have several *problem causes* and several *problem effects*. The holistic identification of problems can usually only be achieved through the participation of all *stakeholders* and additional expertise.

A problem cause is defined as a characteristic of the envisaged innovation that is most likely to cause a *problem*. When identifying the *problem causes*, technical, organizational, and personal characteristics of the innovation or their combination can be taken into account. It is advisable to identify the most likely *problem cause* or most likely *problem causes* and not all possible *problem causes*.

A problem compensation measure describes a solution to eliminate or to reduce the *problem cause*. The solution should be based on changing the characteristics of the envisaged innovation.

A problem effect is defined as a possible negative consequence of a problem.

The severity (S) is a relative ranking for the *problem effect*. The severity can be quantified on a scale ranging from insignificant (1) to catastrophic (10). However, other quantification scales are possible.

The risk priority number (RPN) is calculated as a product of the values severity *(S)*, *probability of occurrence (O)*, and *detectability* (*D)* and is used to prioritize the *causal chains* by means of a ranking order.

A stakeholder is part of the *classification*. The stakeholder is conceptualized as an individual or a group of people directly or indirectly affected by the envisaged innovation. In addition, stakeholders also include individuals or groups of people who can directly or indirectly influence the envisaged innovation. A stakeholder can also not be a natural person (e.g. nature or law) represented by legal entities or interest groups.

The viewing level is part of the *classification*. The viewing levels conceptualize areas in the fields of ELSI, ELSA, or RRI to which usually several *problems* can be assigned. The viewing levels must be formulated in a value-neutral way.

The viewing context includes a description of the envisaged innovation with functions and characteristics as well as the later application.

## Appendix 2: Process Diagram of AMICAI

See Fig. 5.

## Appendix 3: Form Sheet

See Fig. 6.
